# Dose and Response Metrics in Nanotoxicology: Wittmaack Responds to Oberdoerster et al. and Stoeger et al.

**Published:** 2007-06

**Authors:** Klaus Wittmaack

**Affiliations:** GSF–National Research Center for Environment and Health, Institute of Radiation Protection, Neuherberg, Germany, E-mail: wittmaack@gsf.de

In their letters, Oberdörster et al. and Stoeger et al. present some comments on a few out of many issues that I addressed in my reanalysis of literature data on lung inflammatory response to nanoparticle exposure (Wittmaack 2007). I appreciate the opportunity to strengthen and expand my arguments.

I argue that results of nanoparticle toxicology studies should not be interpreted on the basis of the reasoning that the number of surface atoms, relative to all atoms in a (spherical) particle, increases as the inverse of the diameter, *D* (Oberdörster et al. 2005). If the toxicity of an insoluble particle scales with the number of surface atoms, it is the surface area (*A*) that counts, not its ratio to the mass (*M*). Figure 1 shows the size dependence of the specific surface area (*S* = *A/M* = 6/ρ*D*) for TiO_2_ particles [mass density, ρ(anatase) = 3.9 g/cm^3^]. Also presented is an example for the cumulative surface area (∑*A_ae_*) calculated from the mean number concentration of an ambient aerosol (Wittmaack 2002), including extrapolated data for *D* < 10 nm. ∑*A_ae_* decreases rapidly with decreasing *D*, notably for *D* < 100 nm. In contrast, *S_ae_* = ∑*A_ae_/*∑*M_ae_* = ∑*A_ae_/*ρ∑*V_ae_* (ρ = 1.5 g/cm^3^) increases as 1/*D*, for *D* < 200 nm, where *V* is the particle volume. If toxicity is assessed by reference to *S_ae_* rather than to *A_ae_*, the danger of exposure to nanoparticles (e.g., for *D* = 30 nm), compared to fine particles (*D* = 1 μm), is overestimated by a factor of 1,130. By taking the ratio *A/M*, we compare apples (the surface area of insoluble particles) and oranges (the mass of soluble particles).

This type of reasoning in terms of *S_ae_* (Oberdörster et al. 2005) has been used often (Kreyling et al. 2006; Nel et al. 2006 ); Gwinn and Vallyathan (2006) even characterized ultrafine particles (UFPs; i.e., particles with *D* ≤ 100 nm) as “UFPs with larger surface area.”

In their Figure 1, Oberdörster et al. (2005) reproduced some of their own data in two ways: as the number (*n_PMN_*) of lavaged polymorphonuclear leukocytes (PMNs) and as the ratio (*r_P,m_*) of *n_PMN_* to the number (*n_ma_*) of macrophages (*r_P,m_* = *n_PMN_/n_ma_*). To demonstrate that the particle number is not an appropriate dose metric in the special case of TiO_2_, the data could have been presented in a single graph. I found that particle number is a suitable dose metric for differently prepared carbon nanoparticles (Wittmaack 2007). In their letter, Oberdörster et al. use the comparison between *n_PMN_* and *r_P,m_* to argue that “the choice of the response metric is irrelevant.” Data analysis shows that in their study *n_ma_* was essentially constant (10.9 ± 0.5) × 10^6^. Hence, if *n_PMN_* is divided by *n_ma_* ≅ constant, on appropriate scales, the ratio *r_P,m_* looks essentially the same as the *n_PMN_*. Clearly, this result is not proof of the cited assertion.

To explore this issue further, Figure 2 shows a direct comparison of *r_P,m_* with the corresponding fractions *f_P,m_* = *n_PMN_* /(*n_PMN_* + *n_ma_*) = *r_P,m_* */*(1+*r_P,m_*) for the 250-nm TiO_2_ data, according to Oberdörster et al.’s letter. The solid line in Figure 2, derived by linear regression analysis of the *r_P,m_* data, agrees well with previous results (Wittmaack 2007). Further evaluation provided the clue to the issue in question. By converting the *r_P,m_* regression data to fractions *f_P,m_*, I obtained the curve (dashed line), which is clearly nonlinear. Hence, using the *f_P,m_* approach, Oberdörster (2000) converted an existing linear dose–response relationship (for *n_PMN_* or *r_P,m_*) artificially to a dependence that feigns the onset of saturation effects. Therefore, the choice of the response metric is not irrelevant.

Preparing Figure 1 of their letter, Stoeger et al. changed from the right (*n_PMN_*) (Stoeger et al. 2006) to the wrong (*f_P,m_*) response metric. For mice exposed to different types of carbon particles except for those with high carbon content (SootH), I derived from their Figure 1B rather high mean lung masses of 0.287 ± 0.047 g, and even higher values (0.469 ± 0.028 g) for the SootH-exposed animals. The ratio of these two masses (0.61) is the same as that of the ratio *S_BET_*(SootH)/*S_BET_* (SootL). This means that their data were erroneously permuted. Also, the *f_P,m_* carbon particle data are poorly correlated with the original *n_PMN_* data (Stoeger et al. 2006) because the numbers of “lavaged cells,” presumably macrophages, derived from the *n_PMN_* and *f_P,m_* data, differ vastly (i.e., between about 2 × 10^5^ and 3 × 10^6^. Hence, either the *f_P,m_* data in the letter of Stoeger et al. were miscalculated, or *n_ma_* exhibited a biologically unreasonable spread. Furthermore, they include 15 response data for carbon in their letter, but the linear dose–response region contains only 13 (Wittmaack 2007).

In their effort to show that the surface area constitutes a proper all-particle dose metric, Stoeger et al. (2006) discredited their own transmission electron microscopy analysis. Their argument is irrelevant because the spark-generated particles contributed only one data point to a total of 13. Finally, Stoeger et al. do not accept one of the most important points of my article: Carbon particles of different origin exhibit large differences in surface toxicity and, therefore, they cannot be used to identify the best dose metric. Moreover, combining TiO_2_ and carbon data in one graph is not an appropriate comparison.

## Figures and Tables

**Figure 1:**
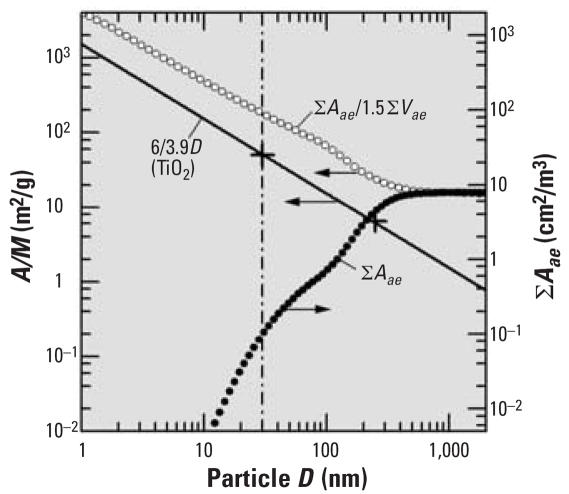
Particle-size dependence of the *A/M* and the ∑*A_ae_*. The straight line relates to TiO_2_ particles, the open and solid circles indicate ambient aerosol particles, and the crosses indicate two BET data. According to Oberdoerster et al.’s letter, the so-called 20-nm TiO_2_ particles may well have been 30 nm in size.

**Figure 2:**
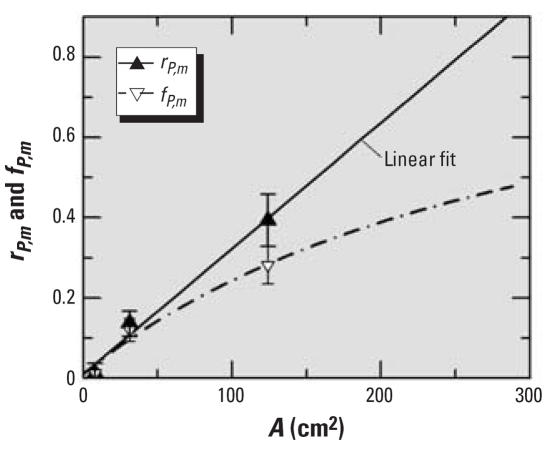
Response of rats to the instillation of 250 nm TiO_2_ particles shown as the *r_P,m_* as reported by Oberdörster et al. in their letter, and the derived *f_P,m_* corresponds to the linear fit through the *r_P,m_* data.

## References

[b1-ehp0115-a00291] Gwinn MR, Vallyathan V (2006). Nanoparticles: health effects—pros and cons. Environ Health Perspect.

[b2-ehp0115-a00291] Kreyling WG, Semmler-Behnke Möller W (2006). Health implications of nanoparticles. J Nanoparticle Res.

[b3-ehp0115-a00291] Nel A, Xia T, Mädler L, Li N (2006). Toxic potential of materials at the nanolevel. Science.

[b4-ehp0115-a00291] Oberdörster G (2000). Toxicology of ultrafine particles: in vivo studies. Phil Trans R Soc Lond.

[b5-ehp0115-a00291] Oberdörster G, Oberdörster E, Oberdörster J (2005). Nanotoxicology: an emerging discipline evolving from studies of ultrafine particles. Environ Health Perspect.

[b6-ehp0115-a00291] Stoeger T, Reinhard C, Takenaka S, Schroeppel A, Karg E, Ritter B (2006). Instillation of six different ultrafine carbon particles indicates a surface area threshold dose for acute lung inflammation in mice. Environ Health Perspect.

[b7-ehp0115-a00291] Wittmaack K (2002). Advanced evaluation of size-differential distributions of aerosol particles. J Aerosol Sci.

[b8-ehp0115-a00291] Wittmaack K 2007 In search of the most relevant parameter for quantifying lung inflammatory response to nanoparticle exposure: particle number, surface area, or what? Environ Health Perspect 115 187 194 10.1289/ehp.9254 [Online 3 October 2006]. 17384763 PMC1831520

